# 4-[(2-Fluoro­phen­yl)amino]-4-oxo­butanoic acid

**DOI:** 10.1107/S1600536808024082

**Published:** 2008-08-06

**Authors:** Farooq Ali Shah, Muhammad Nawaz Tahir, Saqib Ali

**Affiliations:** a Department of Chemistry, Quaid-i-Azam University, Islamabad 45320, Pakistan; b University of Sargodha, Department of Physics, Sargodha, Pakistan

## Abstract

The crystal structure of the title compound, C_10_H_10_FNO_3_, contains dimers of the asymmetric unit, with *R*_2_^2^(8) rings arising from inter­molecular O—H⋯O hydrogen bonding through the carboxyl­ate groups. Adjacent dimeric units are connected to each other through one N—H⋯O and two C—H⋯O inter­molecular hydrogen bonds. C—H⋯O hydrogen bonds involving the aromatic ring and the O atoms of two carboxyl­ate groups form an *R*_3_^3^(7) ring. The crystal structure is further stabilized by C—H⋯F inter­actions, giving rise to a three-dimensional network.

## Related literature

For related literature, see: Bernstein *et al.* (1995 ▶); Shah *et al.* (2008 ▶).
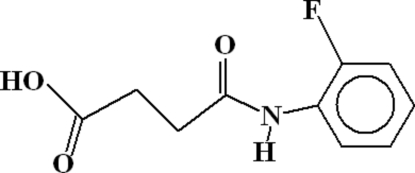


## Experimental

### 

#### Crystal data

C_10_H_10_FNO_3_
*M* *_r_* = 211.19
Monoclinic, 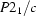

*a* = 4.8054 (3) Å
*b* = 19.0399 (13) Å
*c* = 11.0429 (8) Å
β = 101.821 (3)°
*V* = 988.94 (12) Å^3^
*Z* = 4
Mo *K*α radiation
μ = 0.12 mm^−1^
*T* = 296 (2) K
0.25 × 0.15 × 0.10 mm

#### Data collection

Bruker Kappa APEXII CCD diffractometer
Absorption correction: multi-scan (*SADABS*; Bruker, 2005 ▶) *T*_min_ = 0.975, *T*_max_ = 0.989
11668 measured reflections
2550 independent reflections
1366 reflections with *I* > 2σ(*I*)
*R*_int_ = 0.038

#### Refinement

*R*[*F* ^2^ > 2σ(*F* ^2^)] = 0.050
*wR*(*F* ^2^) = 0.144
*S* = 1.01
2550 reflections
142 parameters
H atoms treated by a mixture of independent and constrained refinement
Δρ_max_ = 0.26 e Å^−3^
Δρ_min_ = −0.20 e Å^−3^

### 

Data collection: *APEX2* (Bruker, 2007 ▶); cell refinement: *APEX2*; data reduction: *SAINT* (Bruker, 2007 ▶); program(s) used to solve structure: *SHELXS97* (Sheldrick, 2008 ▶); program(s) used to refine structure: *SHELXL97* (Sheldrick, 2008 ▶); molecular graphics: *ORTEP-3 for Windows* (Farrugia, 1997 ▶) and *PLATON* (Spek, 2003 ▶); software used to prepare material for publication: *WinGX* (Farrugia, 1999 ▶) and *PLATON*.

## Supplementary Material

Crystal structure: contains datablocks global, I. DOI: 10.1107/S1600536808024082/fj2135sup1.cif

Structure factors: contains datablocks I. DOI: 10.1107/S1600536808024082/fj2135Isup2.hkl

Additional supplementary materials:  crystallographic information; 3D view; checkCIF report

## Figures and Tables

**Table 1:** Hydrogen-bond geometry (Å, °)

*D*—H⋯*A*	*D*—H	H⋯*A*	*D*⋯*A*	*D*—H⋯*A*
O1—H1⋯O2^i^	0.86 (2)	1.81 (2)	2.664 (2)	178 (2)
N1—H1*A*⋯O3^ii^	0.88 (2)	2.04 (2)	2.908 (2)	169.7 (18)
C8—H8⋯O2^iii^	0.93	2.54	3.435 (3)	160
C9—H9⋯O1^iv^	0.93	2.58	3.402 (3)	147
C2—H2*B*⋯F1^v^	0.97	2.61	3.357 (2)	134
C2—H2*A*⋯F1^vi^	0.97	2.82	3.602 (2)	138

Symmetry codes: (i) 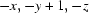; (ii) 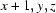; (iii) 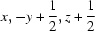; (iv) 
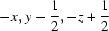
; (v) 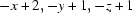; (vi) 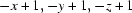.

**Fig. 1.**
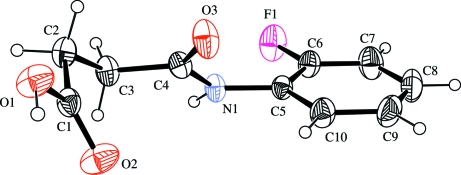
ORTEP-3 for Windows (Farrugia, 1997) drawing of the title compound, C10H10FNO3 with the atom numbering scheme. The thermal ellipsoids are drawn at the 30% probability level. H-atoms are shown by small circles of arbitrary radii.

**Fig. 2.**
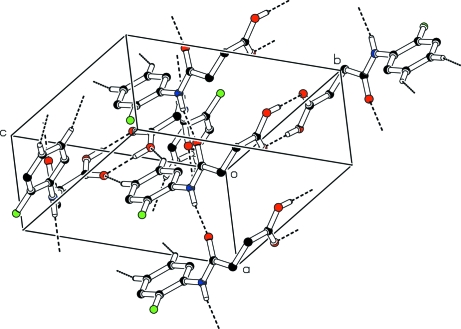
The partial unit cell packing of (I) (Spek, 2003) with only H-atoms which are involved in H-bonding, showing the dimeric nature formig R22(8) ring, forming R33(7) ring through intermolecular H-bonds and the linkage of dimers through H-bonds of N—H···O type.

## supplementary crystallographic information

### Comment

The title compound (I) results from our continuing studies into the synthesis of carboxylic acids having the possibility of coordination with more donor atoms (Shah, *et al.*, 2008). The purpose of synthesizing (I) was to make complexes with various metals and to study the biological activity at large.

The structures of (II) 3-(3,5-dichloroanilinocarbonyl)propionic acid (Shah, *et al.*, 2008) is the best example for comparison of geometry. In (I) the C=O bond distances for carboxylate and carbonyl group have values of (C1=O2: 1.215 (2) Å) and (C4=O3: 1.223 (2) Å) in comparison to 1.219 (3) Å and 1.225 (2) Å, respectively. The C—N bond distances are compareable within experimental errors. The crystal structure of (I) consists of cetro-symmetric dimers forming *R*_2_^2^(8) ring (Bernstein, *et al.*, 1995), through intermolecular H-bonding (Table 1). The adjacent dimers are connected to each other through two C—H···O intermolecular H-bonds forming *R*_3_^3^(7) ring [O—H···O···H—C—C—H···O] as shown in Fig 2. In (I) and (II), there is similarity of H-bonding between the amino and carbonyl group. There are C—H···F interaction also (Table 1) which stabilize the title molecule. The dihedral angle between the aromatic ring (C5—C10) and (C1,C2,C3,O1,O2) have a value of 58.87 (6)°, whereas with (N1,C3,C4,O3) its value is 51.09 (16)°. The value of dihedral angle between (C1,C2,C3,O1,O2) and (N1,C3,C4,O3) is 74.17 (13)°.

### Experimental

2-Fluoroaniline (0.1 mole, 9.56 ml) was dissolved in 30 ml of glacial acetic acid. A solution of succinic anhydride (10 g, 0.1 mole) in 50 ml glacial acetic acid was added and the mixture was stirred overnight. The precipitate which appeared was filtered, washed with distilled water and dried at 313–315 K. The acid was recrystallized from acetone. (Yield: 85%, m.p: 435 K)

### Refinement

The coordinates of H-atom attached with O1 and N1 were refined. The H-atoms attached with C-atoms were positioned geometrically, C—H = 0.93, and 0.97 Å for aromatic and methylene H, and constrained to ride on their parent atoms. The H-atoms were treated as isotropic with *U*_iso_(H) = *xU*_eq_(C, N, O), where *x* = 1.5 for methyl H, and *x* = 1.2 for all other H atoms.

### Crystal data

**Table:**

C_10_H_10_FNO_3_	*F*_000_ = 440
*M**_r_* = 211.19	*D*_x_ = 1.418 Mg m^−^^3^
Monoclinic, *P*2_1_/*c*	Mo *K*α radiation λ = 0.71073 Å
Hall symbol: -P 2ybc	Cell parameters from 2550 reflections
*a* = 4.8054 (3) Å	θ = 2.1–28.7º
*b* = 19.0399 (13) Å	µ = 0.12 mm^−^^1^
*c* = 11.0429 (8) Å	*T* = 296 (2) K
β = 101.821 (3)º	Needle, colorless
*V* = 988.94 (12) Å^3^	0.25 × 0.15 × 0.10 mm
*Z* = 4	

### Data collection

**Table:**

Bruker Kappa APEXII CCD diffractometer	2550 independent reflections
Radiation source: fine-focus sealed tube	1366 reflections with *I* > 2σ(*I*)
Monochromator: graphite	*R*_int_ = 0.038
Detector resolution: 7.4 pixels mm^-1^	θ_max_ = 28.7º
*T* = 296(2) K	θ_min_ = 2.1º
ω scans	*h* = −6→5
Absorption correction: multi-scan(SADABS; Bruker, 2005)	*k* = −25→25
*T*_min_ = 0.975, *T*_max_ = 0.989	*l* = −14→14
11668 measured reflections	

### Refinement

**Table:**

Refinement on *F*^2^	Secondary atom site location: difference Fourier map
Least-squares matrix: full	Hydrogen site location: inferred from neighbouring sites
*R*[*F*^2^ > 2σ(*F*^2^)] = 0.050	H atoms treated by a mixture of independent and constrained refinement
*wR*(*F*^2^) = 0.145	*w* = 1/[σ^2^(*F*_o_^2^) + (0.0685*P*)^2^ + 0.1019*P*] where *P* = (*F*_o_^2^ + 2*F*_c_^2^)/3
*S* = 1.01	(Δ/σ)_max_ < 0.001
2550 reflections	Δρ_max_ = 0.26 e Å^−^^3^
142 parameters	Δρ_min_ = −0.20 e Å^−^^3^
Primary atom site location: structure-invariant direct methods	Extinction correction: none

### Special details

**Table:**

Geometry. Bond distances, angles *etc*. have been calculated using the rounded fractional coordinates. All su's are estimated from the variances of the (full) variance-covariance matrix. The cell e.s.d.'s are taken into account in the estimation of distances, angles and torsion angles
Refinement. Refinement of *F*^2^ against ALL reflections. The weighted *R*-factor *wR* and goodness of fit *S* are based on *F*^2^, conventional *R*-factors *R* are based on *F*, with *F* set to zero for negative *F*^2^. The threshold expression of *F*^2^ > σ(*F*^2^) is used only for calculating *R*-factors(gt) *etc*. and is not relevant to the choice of reflections for refinement. *R*-factors based on *F*^2^ are statistically about twice as large as those based on *F*, and *R*- factors based on ALL data will be even larger.

### Fractional atomic coordinates and isotropic or equivalent isotropic displacement parameters (Å^2^)

**Table:**

	*x*	*y*	*z*	*U*_iso_*/*U*_eq_	
F1	1.0488 (3)	0.33085 (7)	0.62407 (12)	0.0777 (5)	
O1	0.0318 (3)	0.56812 (8)	0.11407 (14)	0.0633 (6)	
O2	0.2963 (3)	0.47537 (8)	0.09700 (14)	0.0675 (6)	
O3	0.2983 (3)	0.42965 (8)	0.37996 (15)	0.0638 (6)	
N1	0.7391 (3)	0.38295 (8)	0.40847 (14)	0.0419 (5)	
C1	0.2503 (4)	0.52781 (10)	0.15251 (18)	0.0422 (6)	
C2	0.4378 (4)	0.55192 (10)	0.26901 (19)	0.0484 (6)	
C3	0.6657 (4)	0.49982 (10)	0.32502 (19)	0.0469 (6)	
C4	0.5482 (3)	0.43475 (10)	0.37282 (16)	0.0400 (6)	
C5	0.6718 (4)	0.31736 (9)	0.45548 (17)	0.0381 (6)	
C6	0.8291 (4)	0.29162 (10)	0.56401 (18)	0.0470 (6)	
C7	0.7714 (5)	0.22849 (12)	0.6121 (2)	0.0671 (8)	
C8	0.5497 (5)	0.18915 (12)	0.5515 (2)	0.0681 (9)	
C9	0.3881 (5)	0.21314 (11)	0.4428 (2)	0.0643 (8)	
C10	0.4499 (4)	0.27661 (11)	0.39432 (19)	0.0542 (7)	
H1	−0.070 (5)	0.5532 (12)	0.046 (2)	0.0759*	
H1A	0.916 (4)	0.3921 (10)	0.4045 (18)	0.0503*	
H2A	0.32139	0.56196	0.32903	0.0581*	
H2B	0.52867	0.59543	0.25277	0.0581*	
H3A	0.77241	0.48655	0.26294	0.0563*	
H3B	0.79632	0.52230	0.39250	0.0563*	
H7	0.88285	0.21248	0.68592	0.0805*	
H8	0.50824	0.14620	0.58383	0.0817*	
H9	0.23582	0.18650	0.40136	0.0771*	
H10	0.34085	0.29209	0.31962	0.0650*	

### Atomic displacement parameters (Å^2^)

**Table:**

	*U*^11^	*U*^22^	*U*^33^	*U*^12^	*U*^13^	*U*^23^
F1	0.0646 (8)	0.0765 (9)	0.0763 (9)	−0.0227 (7)	−0.0221 (7)	0.0104 (7)
O1	0.0610 (10)	0.0615 (10)	0.0597 (10)	0.0270 (8)	−0.0055 (7)	−0.0027 (7)
O2	0.0655 (10)	0.0638 (10)	0.0645 (10)	0.0308 (8)	−0.0073 (8)	−0.0113 (8)
O3	0.0265 (7)	0.0653 (10)	0.1013 (12)	0.0009 (6)	0.0170 (7)	0.0277 (8)
N1	0.0247 (7)	0.0430 (9)	0.0571 (10)	−0.0037 (7)	0.0065 (7)	0.0100 (7)
C1	0.0396 (10)	0.0384 (11)	0.0494 (11)	0.0076 (9)	0.0113 (9)	0.0120 (9)
C2	0.0434 (10)	0.0409 (10)	0.0591 (12)	−0.0036 (9)	0.0060 (9)	0.0057 (9)
C3	0.0335 (9)	0.0497 (11)	0.0552 (12)	−0.0062 (8)	0.0034 (9)	0.0103 (9)
C4	0.0278 (8)	0.0474 (11)	0.0433 (10)	−0.0045 (8)	0.0038 (7)	0.0057 (8)
C5	0.0311 (9)	0.0373 (10)	0.0465 (10)	−0.0036 (8)	0.0094 (8)	0.0014 (8)
C6	0.0396 (10)	0.0451 (11)	0.0525 (11)	−0.0081 (9)	0.0007 (9)	0.0020 (9)
C7	0.0680 (15)	0.0628 (15)	0.0654 (14)	−0.0041 (13)	0.0018 (12)	0.0211 (12)
C8	0.0742 (16)	0.0465 (12)	0.0866 (18)	−0.0138 (12)	0.0233 (14)	0.0113 (12)
C9	0.0606 (13)	0.0506 (13)	0.0804 (16)	−0.0227 (11)	0.0114 (12)	−0.0102 (12)
C10	0.0496 (12)	0.0540 (13)	0.0551 (12)	−0.0146 (10)	0.0014 (9)	0.0000 (10)

### Geometric parameters (Å, °)

**Table:**

F1—C6	1.351 (2)	C6—C7	1.365 (3)
O1—C1	1.300 (2)	C7—C8	1.361 (3)
O2—C1	1.215 (2)	C8—C9	1.368 (3)
O3—C4	1.223 (2)	C9—C10	1.379 (3)
O1—H1	0.86 (2)	C2—H2A	0.9700
N1—C4	1.350 (2)	C2—H2B	0.9700
N1—C5	1.415 (2)	C3—H3A	0.9700
N1—H1A	0.88 (2)	C3—H3B	0.9700
C1—C2	1.484 (3)	C7—H7	0.9300
C2—C3	1.513 (3)	C8—H8	0.9300
C3—C4	1.502 (3)	C9—H9	0.9300
C5—C10	1.378 (3)	C10—H10	0.9300
C5—C6	1.370 (3)		
			
F1···N1	2.723 (2)	C6···C9^vii^	3.566 (3)
F1···C2^i^	3.357 (2)	C9···O1^viii^	3.402 (3)
F1···H1A	2.648 (19)	C9···C6^v^	3.566 (3)
F1···H2A^ii^	2.8200	C10···O3	3.000 (3)
F1···H2B^i^	2.6100	C1···H1^iii^	2.68 (2)
O1···O2^iii^	2.664 (2)	C1···H3A^v^	2.9200
O1···C9^iv^	3.402 (3)	C4···H10	2.9100
O2···C1^iii^	3.396 (2)	H1···O2^iii^	1.81 (2)
O2···C4	3.135 (2)	H1···C1^iii^	2.68 (2)
O2···O1^iii^	2.664 (2)	H1···H1^iii^	2.42 (3)
O3···C3^v^	3.262 (2)	H1A···F1	2.648 (19)
O3···C1	3.101 (3)	H1A···O3^vii^	2.04 (2)
O3···N1^v^	2.908 (2)	H1A···H3A	2.3900
O3···C10	3.000 (3)	H1A···H3B	2.5400
O1···H9^iv^	2.5800	H2A···O3	2.5900
O1···H3A^v^	2.7400	H2A···F1^ii^	2.8200
O2···H8^vi^	2.5400	H2B···F1^i^	2.6100
O2···H1^iii^	1.81 (2)	H3A···O1^vii^	2.7400
O2···H3A	2.6300	H3A···O2	2.6300
O3···H1A^v^	2.04 (2)	H3A···O3^vii^	2.8100
O3···H2A	2.5900	H3A···C1^vii^	2.9200
O3···H3A^v^	2.8100	H3A···H1A	2.3900
O3···H10	2.7200	H3B···H1A	2.5400
O3···H3B^ii^	2.8000	H3B···O3^ii^	2.8000
N1···F1	2.723 (2)	H7···H10^ix^	2.3900
N1···O3^vii^	2.908 (2)	H8···O2^x^	2.5400
C1···O3	3.101 (3)	H9···O1^viii^	2.5800
C1···O2^iii^	3.396 (2)	H10···O3	2.7200
C2···F1^i^	3.357 (2)	H10···C4	2.9100
C3···O3^vii^	3.262 (2)	H10···H7^xi^	2.3900
C4···O2	3.135 (2)		
			
C1—O1—H1	111.6 (16)	C8—C9—C10	120.3 (2)
C4—N1—C5	124.00 (15)	C5—C10—C9	120.65 (19)
C4—N1—H1A	116.6 (13)	C1—C2—H2A	109.00
C5—N1—H1A	119.3 (13)	C1—C2—H2B	109.00
O1—C1—O2	122.59 (18)	C3—C2—H2A	109.00
O1—C1—C2	114.01 (17)	C3—C2—H2B	109.00
O2—C1—C2	123.40 (18)	H2A—C2—H2B	108.00
C1—C2—C3	114.31 (16)	C2—C3—H3A	109.00
C2—C3—C4	113.08 (16)	C2—C3—H3B	109.00
N1—C4—C3	115.05 (14)	C4—C3—H3A	109.00
O3—C4—C3	122.21 (17)	C4—C3—H3B	109.00
O3—C4—N1	122.74 (17)	H3A—C3—H3B	108.00
N1—C5—C6	120.67 (17)	C6—C7—H7	120.00
N1—C5—C10	121.94 (17)	C8—C7—H7	120.00
C6—C5—C10	117.39 (17)	C7—C8—H8	120.00
C5—C6—C7	122.39 (19)	C9—C8—H8	120.00
F1—C6—C5	117.86 (17)	C8—C9—H9	120.00
F1—C6—C7	119.76 (18)	C10—C9—H9	120.00
C6—C7—C8	119.6 (2)	C5—C10—H10	120.00
C7—C8—C9	119.6 (2)	C9—C10—H10	120.00
			
C5—N1—C4—O3	1.1 (3)	N1—C5—C6—C7	−179.78 (19)
C5—N1—C4—C3	−179.64 (16)	C10—C5—C6—F1	179.37 (17)
C4—N1—C5—C6	−129.3 (2)	C10—C5—C6—C7	−0.5 (3)
C4—N1—C5—C10	51.4 (3)	N1—C5—C10—C9	−179.55 (19)
O1—C1—C2—C3	170.92 (17)	C6—C5—C10—C9	1.2 (3)
O2—C1—C2—C3	−9.5 (3)	F1—C6—C7—C8	179.9 (2)
C1—C2—C3—C4	−67.8 (2)	C5—C6—C7—C8	−0.3 (3)
C2—C3—C4—O3	−9.2 (3)	C6—C7—C8—C9	0.3 (4)
C2—C3—C4—N1	171.57 (16)	C7—C8—C9—C10	0.4 (4)
N1—C5—C6—F1	0.1 (3)	C8—C9—C10—C5	−1.1 (3)

Symmetry codes: (i) −*x*+2, −*y*+1, −*z*+1; (ii) −*x*+1, −*y*+1, −*z*+1; (iii) −*x*, −*y*+1, −*z*; (iv) −*x*, *y*+1/2, −*z*+1/2; (v) *x*−1, *y*, *z*; (vi) *x*, −*y*+1/2, *z*−1/2; (vii) *x*+1, *y*, *z*; (viii) −*x*, *y*−1/2, −*z*+1/2; (ix) *x*+1, −*y*+1/2, *z*+1/2; (x) *x*, −*y*+1/2, *z*+1/2; (xi) *x*−1, −*y*+1/2, *z*−1/2.

### Hydrogen-bond geometry (Å, °)

**Table:**

*D*—H···*A*	*D*—H	H···*A*	*D*···*A*	*D*—H···*A*
O1—H1···O2^iii^	0.86 (2)	1.81 (2)	2.664 (2)	178 (2)
N1—H1A···O3^vii^	0.88 (2)	2.04 (2)	2.908 (2)	169.7 (18)
C8—H8···O2^x^	0.93	2.54	3.435 (3)	160
C9—H9···O1^viii^	0.93	2.58	3.402 (3)	147
C2—H2B···F1^i^	0.97	2.61	3.357 (2)	134
C2—H2A···F1^ii^	0.97	2.82	3.602 (2)	138

Symmetry codes: (iii) −*x*, −*y*+1, −*z*; (vii) *x*+1, *y*, *z*; (x) *x*, −*y*+1/2, *z*+1/2; (viii) −*x*, *y*−1/2, −*z*+1/2; (i) −*x*+2, −*y*+1, −*z*+1; (ii) −*x*+1, −*y*+1, −*z*+1.
